# Numerical Study of Low-Velocity Impact Response of a Fiber Composite Honeycomb Sandwich Structure

**DOI:** 10.3390/ma16155482

**Published:** 2023-08-05

**Authors:** Zhou Wen, Ming Li

**Affiliations:** 1 College of Mechanical Engineering, Xi’an University of Science and Technology, Xi’an 710054, China; 2 College of Media Communication, Dongguan Polytechnic, Dongguan 523808, China; 3 College of Science, Xi’an University of Science and Technology, Xi’an 710054, China; limxust@xust.edu.cn

**Keywords:** low-velocity impact, heterogeneous sandwich structure, fiber-reinforced, finite element analysis

## Abstract

Engineering applications for honeycomb sandwich structures (HSS) are well recognized. Heterogeneous structures have been created using polyetheretherketone (PEEK) material, glass fiber-reinforced PEEK (GF-PEEK), and carbon fiber-reinforced PEEK (CF-PEEK) to further enhance the load-carrying capacity, stiffness, and impact resistance of HSS. In this study, we investigated the low-velocity impact response of HSS using numerical simulation. Our findings demonstrate that the choice of construction material significantly affects the impact resistance and structural stability of the HSS. We found that using fiber-reinforced PEEK significantly enhances the impact resistance of the overall structure, with GF-PEEK identified as the more appropriate face sheet material for the composite HSS based on a comparative study of load–displacement curves. Analysis of the plastic deformation of the honeycomb core, in combination with the stress and strain distribution of the composite HSS after low-velocity impact, indicates that CF-PEEK face sheets cause more noticeable damage to the core, resulting in evident plastic deformation. Additionally, we discovered that the use of fiber-reinforced materials effectively reduces deflection during low-velocity dynamic impact, particularly when both the face sheet and honeycomb core of the HSS are composed of the same fiber-reinforced PEEK material. These results provide valuable insights into the design and optimization of composite HSS for impact resistance applications.

## 1. Introduction

Honeycomb sandwich structures (HSS) are widely used as a weight-saving material in automobiles, aircraft, ships, and trains [1,2,3] because of their excellent energy absorption capacity, high flexural shear stiffness, and low weight-to-force ratio [4,5]. These structures are inevitably subject to impact loads during manufacturing, installation, maintenance, and service life. For this reason, it is essential to enhance their dynamic impact response and impact resistance under low-velocity impact [6,7,8]. In light of this, this study aims to determine the best materials for strengthening the impact resistance of heterogeneous HSS created using heterogeneous materials by analyzing how they respond to impacts.

The stiffness and structural stability of HSS are two essential performance parameters of these structures, especially these structures that suffer from vibration, impact, and falls [9,10,11,12]. Significant investigations have been conducted using experimental and numerical methods to better understand their dynamic mechanical features, including compression, impact resistance, and energy absorption capacity [13,14,15]. The impact performance of HSS composites is significantly influenced by the honeycomb materials and geometric dimensions [16,17,18]. Changing the material or geometric configuration parameters of HSS are two typical techniques for improving their mechanical properties: for instance, choosing different materials of face sheets, changing the face sheet thickness, and optimizing core structure [7,8,19,20,21]. In light of this, the performance of heterogeneous HSS made with varied materials for the face sheets and core is the primary focus of this research.

Currently, the study on the improvement of dynamic impact performance by design or optimization of HSS has been significantly established by many researchers. Novel HSS, such as honeycomb-filled structures [22,23,24], embedded HSS [25,26], and tandem honeycombs [27,28], have been proposed to enhance mechanical properties. For instance, tandem hexagonal HSS was exposed to axial compressive loads in a series of studies by Wang et al. [27]. They found that loading the tandem honeycomb into multi-cell tubes improved the mechanical behavior. A unique anisotropic hexagonal honeycomb with walls composed of triangular or Kagome honeycombs was proposed by Sun et al. [29]. This HSS significantly increased the in-plane stiffness compared with the conventional HSS. Sabah et al. [8] studied an impact-loaded bio-inspired honeycomb sandwich beam (BHSB) with four primary layers: carbon fiber-reinforced plastic top and bottom sheets, sandwiching rubber, and aluminum honeycomb cores. Compared with standard honeycomb sandwich beams (HSB), the BHSB bottom sheet had a slightly damaged area and surfed consistently with lower stresses and a stronger impact. Based on the periodic region of the dactyl club, Han et al. [30] created a dactyl-inspired sandwich honeycomb (DSH) utilizing a unidirectional carbon fiber and aluminum honeycomb. The quasi-isotropic helicoidal arrangement of the carbon fiber effectively improved the impact resistance and bending energy absorption of DSH. The choice of materials is a crucial aspect that can significantly impact the performance of HSS and attract the interest of researchers. In this regard, Florence et al. [31] conducted experimentally and numerically investigated the dynamic impact behavior of hybrid fiber HSS filled with various energy-absorbing materials in their core. The result shows that the honeycomb sandwich structure with a polyurethane foam filled in the core performs better energy absorption capacity. Crupi et al. [20] compared the peak load and energy absorption of an aluminum honeycomb sandwich and a glass fiber-reinforced plastic (GFRP) panel; they reported that using a GFRP panel as the face sheets of an aluminum honeycomb sandwich can increase energy absorption and bearing capacity. The impact response of carbon fiber-reinforced plastic (CFRP) sandwich panels with various parameterized honeycomb cores was compared by He et al. [17]. The addition of honeycomb filling to the CFRP structures improved their impact resistance, resulting in higher energy absorption and lower peak loads during impact. In a different investigation, quasi-static axial compression experiments were used by Liu et al. [32] to examine the mechanical characteristics of CFRP square tubes packed with honeycomb. The maximum load and absorbed energy of the filled tubes increased by more than 10% compared with those of the empty CFRP tubes.

With excellent mechanical and tribological properties, PEEK has been increasingly employed in different industries, including aerospace, automotive, rail transit, medical, etc. In recent years, many researchers have begun to focus on the mechanical properties of PEEK. Wang et al. [33] investigated the bending and compression properties of PEEK influenced by 3D-printing parameters, and the result shows that the nozzle diameter is the most important factor affecting the bending and compression performance. Wang et al. [34] employed tensile experiments to compare the tribological and mechanical properties of neat PEEK with short basalt fiber (BF) reinforced PEEK, finding that the ultimate tensile strength of BF PEEK (25 wt.% BF) is much higher than that of neat PEEK, reaching 150 MPa. Gummadi et al. [35] applied an experimental and numerical investigation of PEEK scaffolds subjected to quasi-static compression tests. They observed that a PEEK scaffold with a 300 μm pore size performs the best compressive resistance ability, and the maximum stress is distributed along the longitudinal axis of the scaffold core under compressive load. Arif et al. [36] experimentally investigated the tensile properties and dynamic mechanical properties of neat PEEK, graphene nanoplatelets (GNP), and carbon nanotubes (CNT) reinforced PEEK composites. The result showed that Young’s and storage moduli increased by 66% and 77% for GNP nanocomposites (5 wt.% GNP) and by 20% and 23% for CNT nanocomposites (3 wt.% CNT) in comparison to neat PEEK.

While the static or quasi-static compressive response of PEEK has been extensively studied, there is a lack of research on the dynamic impact behavior of solid PEEK and cellular PEEK. Additionally, there is no literature available on the experimental or numerical investigation of the dynamic impact behavior of cellular PEEK. To address this gap in the literature, we designed PEEK, CF-PEEK, and GF-PEEK as the materials for the face sheet and honeycomb core of an HSS. Finite element methods (FEM) were applied to analyze the stress distribution and deformation mode of the specimens during the dynamic response of the fiber composite honeycomb sandwich structure. Peak load and contact energy of heterogeneous HSS were predicted theoretically.

## 2. Numerical Simulation

### 2.1. Composite Honeycomb Sandwich Structure Design

**Figure 1:**
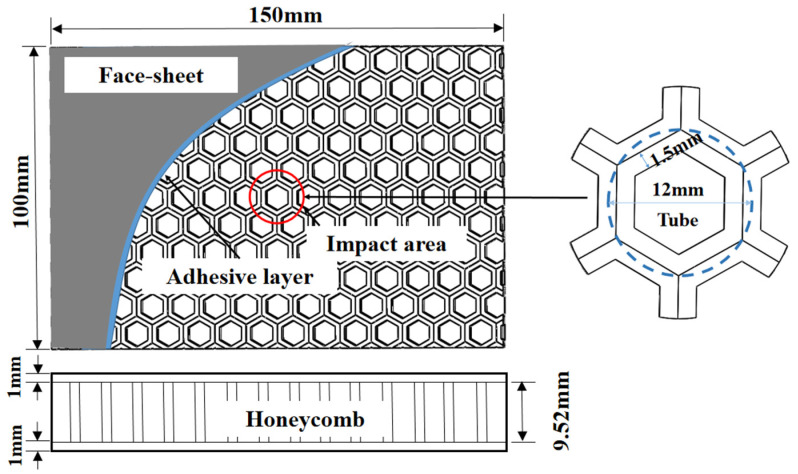
Schematic diagram and geometrical parameters of the specimen.

The present study describes the application of a composite HSS design for numerically calculated drop weight testing, as depicted in Figure 1. The specimens were fixed to a support plate using four clamping devices, which were set to a fully constrained state (i.e., the displacement in the X, Y, and Z directions, as well as the rotation along the X, Y, and Z axis). The support plate featured a 150 mm × 100 mm hole in the center and was presumed to be a rigid base plate. A hemispherical impactor with a diameter of 10 mm was used to impact the specimens with an impact energy of 10 J and a corresponding impact velocity of 1.37 m/s. After the first impact, the impactor rebounded. Based on experimental confirmation by Liu et al. [32], we assumed that the impactor was a rigid base plate with negligible mass influence on the impact process when the impact energy was constant. The impact zone was at the center of the specimen, as indicated by the blue dashed circle in Figure 1.

### 2.2. Mesh Generation, Boundary, and Loading Conditions

**Figure 2:**
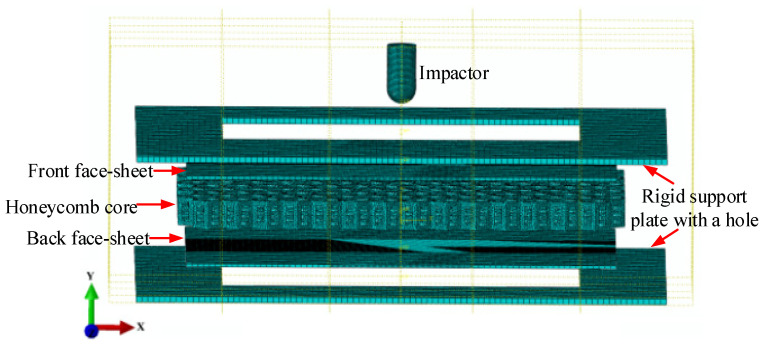
Finite element model of metal honeycomb core sandwich plate subjected to low-velocity impact.

In this section, we provide the numerical models created to analyze the stress distribution and deformation of specimens under the impact of a dropping object. The models were developed using ABAQUS/Explicit (Dassault Systèmes Simulia Corp., Providence, RI, USA), with the dimension parameters of the tested specimens shown in Figure 1. To replicate the thin walls of the honeycomb core, a wall thickness of 1.5 mm was selected. The drop weight impactor and support plate were constructed using discrete rigid parts, with the impactor consisting of 3528 S4R elements. Both support plates were meshed using 3930 CPE8 elements. The honeycomb core was modeled using free meshing, resulting in a model with 354,089 CPE8 elements. The face sheets were filled with 15,300 CPE8 elements and were locally meshed with a higher quality of 0.5 mm around the impact area. Figure 2 illustrates the models developed for the honeycomb core, drop weight impactor, face sheets, and support plate.

As depicted in Figure 2, the back face sheets were likewise attached to the rigid support plate because four clamping devices secured the specimen to the support plate. The kinematic contact method was applied between the impactor and the front face sheet, and the ABAQUS penalty was selected as the contact property. The coefficient of friction was fixed to 0.2 for widespread contact [30,37].

### 2.3. Composite Honeycomb Sandwich Structure Material Design

Table 1 lists the material characteristics of the PEEK, CF-PEEK, and GF-PEEK that were utilized to make the honeycomb core and face sheets, with glass fiber and carbon fiber making up 10% of the total contents of the GF-PEEK and CF-PEEK composites, respectively. As for the three materials (i.e., PEEK, CF-PEEK, and GF-PEEK), the constitutive equation of the three materials conforms to the elastoplastic constitutive relationship. Additionally, the shear failure strain of 0.47 was chosen as the face failure sheet’s criterion [38]. Due to the fiber-reinforced PEEK material’s resistance to strain rate under low-velocity impact loads, the sandwich structure’s strain rate effect was ignored.

In this research, the materials for the face sheet and honeycomb core of the honeycomb sandwich structure were PEEK, and 10% fiber-reinforced PEEK composites (CF-PEEK, GF-PEEK), respectively. The numerical simulation examined a total of nine distinct categories of the honeycomb sandwich structure with various material combinations, which has been summarized in Table 2.

## 3. Results and Discussion

### 3.1. Load–Displacement Curves

**Figure 3:**
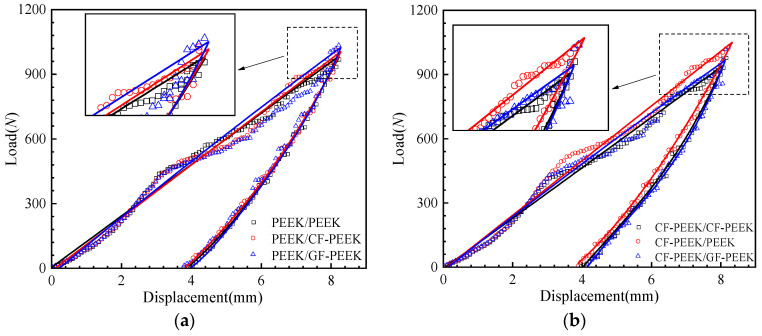

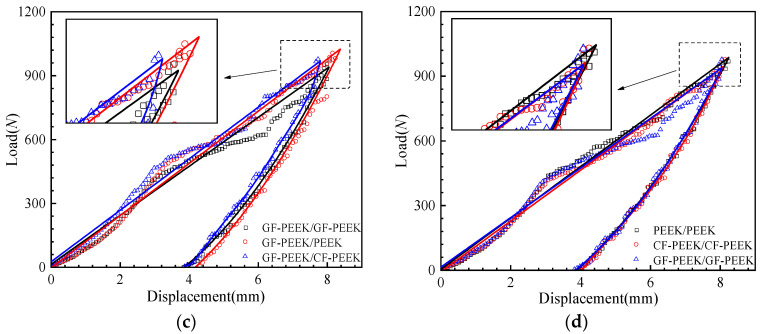
Impact load–displacement curves (**a**) PEEK panels structure; (**b**) CF-PEEK panels structure; (**c**) GF-PEEK panels structure; and (**d**) comparison of the homogeneous material structure.

The low-velocity impact response of the fiber composite HSS with various material combinations was numerically analyzed by finite elements. Each group of HSS panels used the same material, while the honeycomb core material varied. The load–displacement curves for the fiber composite HSS under the impact energy 10 J are given in Figure 3. Among them, Figure 3a–c illustrates PEEK, CF-PEEK, and GF-PEEK, respectively, as panel materials for the HSS. In contrast, Figure 3d compares the homogeneous material for the HSS. Two sections make up the load–displacement curve for the honeycomb sandwich structure: (I) The pressing punch stage: the displacement of the impactor is obviously proportional to the load at this stage, as seen in Figure 3. The HSS will enter the plastic deformation phase as the impactor displacement increases. As the impactor displacement rises, the honeycomb sandwich structure will reach the plastic deformation stage. The description of the structural stress distribution shows that the plastic strain is mainly concentrated on the front panel and honeycomb core. (II) The rebound stage: During this phase, the impactor rebounded throughout this stage, and the contact force rapidly dropped until the punch left the front panel and the contact force was nil.

As illustrated in Figure 3a, when PEEK is used as the front face sheet material of the structure, the honeycomb sandwich structure with GF-PEEK as the core material has to withstand an impact with a relatively high impact load while still having the same impact energy. Additionally, the three material combinations of the HSS depicted in Figure 3a have similar maximum impact displacements during the impact process. It indicated that the impact resistance of the HSS with PEEK as the front face sheet material is approximately 2.25% higher than that of the structure with CF-PEEK as the front face sheet material. The CF-PEEK/PEEK combination also resulted in a higher impact displacement as compared to the other two material combinations, as illustrated in Figure 3b. This shows that the damage caused by the CF-PEEK/PEEK combination is approximately 3.20% more severe compared to the other two material combinations. Similarly, GF-PEEK as a front face sheet material of the structure demonstrates that PEEK as the honeycomb core material can withstand increased impact load and displacement, as illustrated in Figure 3c. Consequently, the GF-PEEK/PEEK combination caused more extensive damage under the same impact energy conditions. The appeals analysis shows that the damage behavior of PEEK employed as the honeycomb core of the structure will be more severe when fiber-reinforced PEEK is used as the front face sheet material. These findings are consistent with previous studies that have reported the superior impact resistance of PEEK composites due to their high strength-to-weight ratio and excellent mechanical properties [42]. Moreover, using honeycomb structures in composite materials has been demonstrated to enhance their energy absorption capability and mechanical properties [43,44].

The load–displacement curves of a homogenous HSS made of three materials are displayed in Figure 3d. It is evident that a structure made of PEEK will result in a higher impact load and impact displacement during the impact process when compared to a structure made of fiber-reinforced PEEK. This demonstrates that the impact load and displacement of the structure made of PEEK are approximately 1.87% and 1.26% higher, respectively, than those of the structure made of fiber-reinforced PEEK. Therefore, combining PEEK and honeycomb structures has excellent potential for developing high-performance and lightweight materials for various engineering applications.

### 3.2. Stress and Strain Distribution

**Figure 4:**
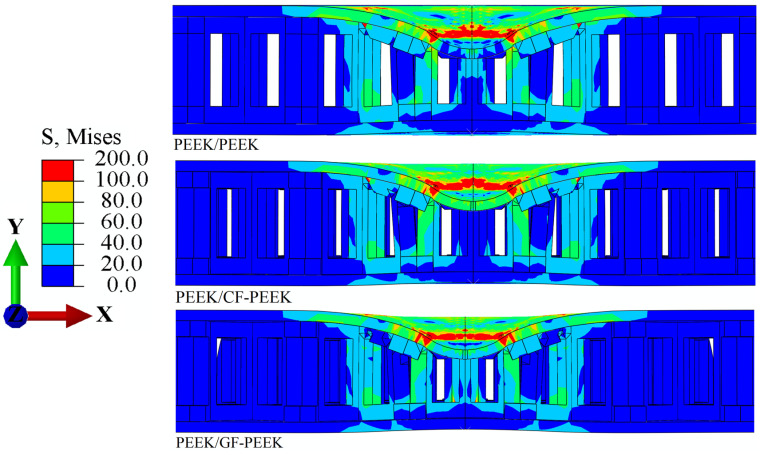
Mises stress *σ*_m_ nephogram cross-section images of the PEEK panel’s structure.

**Figure 5:**
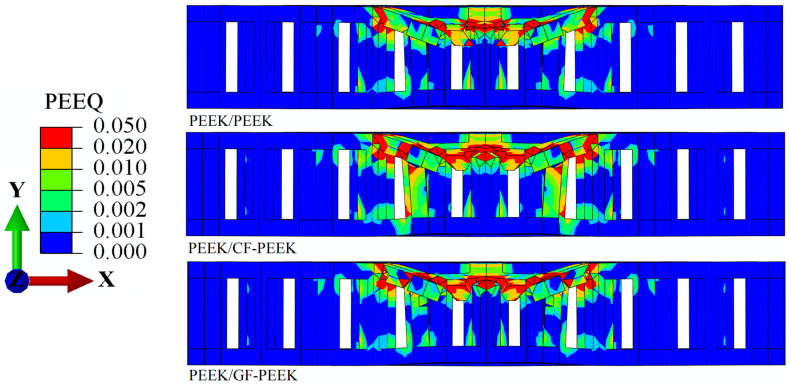
Equivalent plastic strain *ε*_eq_ nephogram cross-section images of the PEEK panel’s structure.

Upon using PEEK as the front face sheet material of HSS, the Mises stress *σ*_m_ and the equivalent plastic strain *ε*_eq_ of the structure’s honeycomb core are illustrated in Figure 4 and Figure 5, respectively. The stress and strain distributions obtained from the numerical simulation showed that the plastic strain was mainly concentrated in the front face sheet and honeycomb core during the impact process at an energy level of 10 J. Specifically, hemispherical defects are left in the impact region of the front face sheet once the impact behavior is complete, as shown in Figure 5. Compared to the hollow part in contact with the core, the part in contact with the honeycomb core experiences a significantly higher stress level. Similarly, the HSS experienced a higher stress level compared to the front face sheets under the same impact energy, while the back face sheets exhibited the opposite effect. However, the back face sheets showed the opposite effect. These observations are consistent with previous studies that have reported the concentration of plastic deformation and damage in the front face sheet and honeycomb core of HSS under impact loading conditions [45]. Moreover, the hemispherical defects observed in the impact region of the front face sheet indicate the occurrence of local material failure and damage. This highlights the importance of selecting appropriate front face sheet materials with high-impact resistance to protect the honeycomb core and avoid catastrophic failure of the structure under impact loading.

Compared to PEEK or GF-PEEK, it is observed in Figure 4 that the stress is significantly higher when CF-PEEK is a front face sheet material of the structure. This result provides additional evidence in favor of the load–displacement curve shown in Figure 3a. When PEEK is used as a front face sheet and CF-PEEK is used as a honeycomb core material, a substantial impact load is generated during the impact process. This is supported by the comparable plastic deformation of the honeycomb core structure observed in Figure 5. Specifically, it is noteworthy that the honeycomb core structure just below the dynamic impact area shows significantly greater plastic deformation when CF-PEEK is employed as the honeycomb core material.

**Figure 6:**
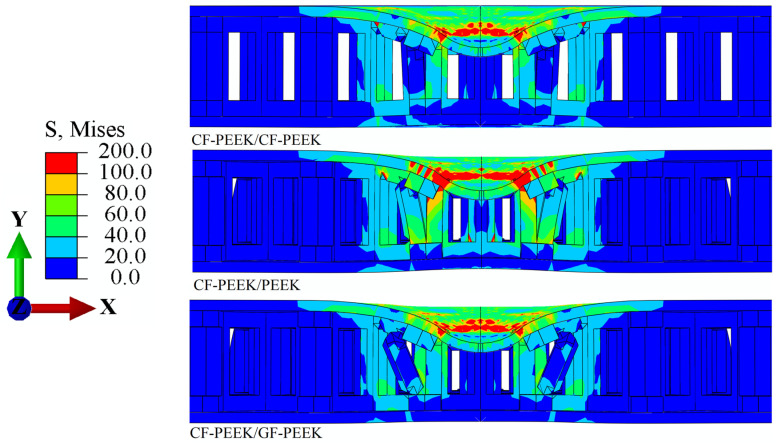
Mises stress *σ*_m_ nephogram cross-section images of the CF-PEEK panel’s structure.

**Figure 7:**
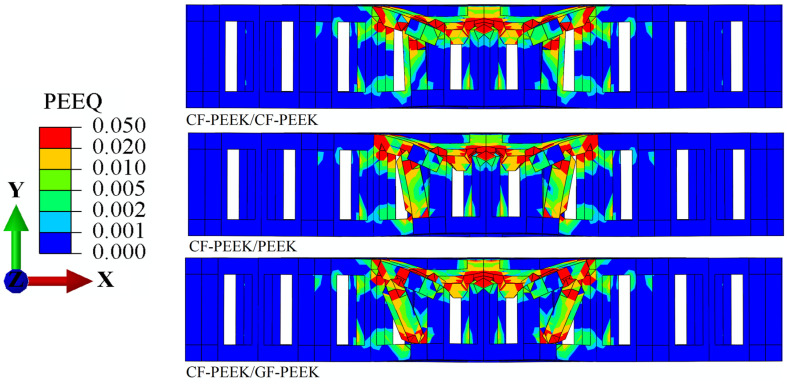
Equivalent plastic strain *ɛ*_eq_ nephogram cross-section images of the CF-PEEK panel’s structure.

Figure 6 and Figure 7 show the distribution of Mises stress *σ*_m_ and the equivalent plastic strain *ε*_eq_ in the HSS when the CF-PEEK is used as the front face sheet material, according to the structural stress distribution of the three materials used as the honeycomb core in Figure 6. It is observed that when PEEK is used as the honeycomb core material, the stress concentration in the impact region of the front face sheet is 1.05% higher than that in CF-PEEK and GF-PEEK. This result is consistent with previous studies that have reported the superior mechanical properties of CF-PEEK and GF-PEEK compared to PEEK [46]. This finding further supports the load–displacement curve shown in Figure 3b, indicating that a significant impact load is generated during the impact process of the honeycomb sandwich structure.

**Figure 8:**
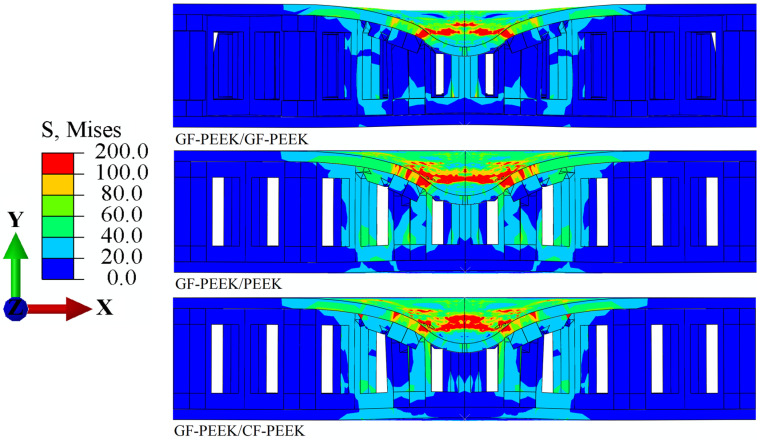
Mises stress *σ*_m_ nephogram cross-section images of the GF-PEEK panel’s structure.

**Figure 9:**
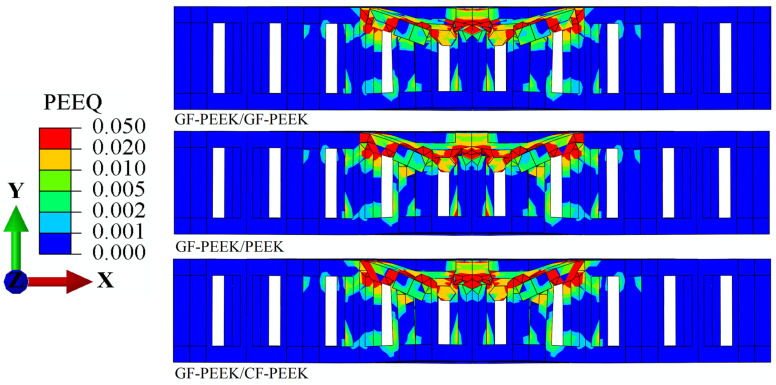
Equivalent plastic strain *ε*_eq_ nephogram cross-section images of the GF-PEEK panel’s structure.

Additionally, it can be seen that the CF-PEEK face sheet damages the honeycomb core is 2.51% greater when GF-PEEK or PEEK is used as the honeycomb core material in Figure 5 and Figure 7. The equivalent plastic strain of the honeycomb core, shown in Figure 7, reveals that, when subjected to the same impact energy, the CF-PEEK material of the face sheet and honeycomb core is relatively more structurally stable than the GF-PEEK and PEEK material of the honeycomb core. This result is supported by previous studies highlighting the superior mechanical properties of CF-PEEK in terms of impact resistance and structural stability [47]. It indicated that the CF-PEEK face sheet damages the honeycomb core more visibly and causes more obvious plastic deformation. The Mises stress *σ*_m_ and equivalent plastic strain *ε*_eq_ for the honeycomb sandwich construction with GF-PEEK as the front face sheet material are depicted in Figure 8 and Figure 9, respectively. It is observed from Figure 8 that the utilization of GF-PEEK as the face sheet material and CF-PEEK as the honeycomb core structural material produces a greater stress concentration distribution as compared to when GF-PEEK is used as the honeycomb core material. On the other hand, the GF-PEEK used as the honeycomb core material results in the smallest stress concentration region. According to the distribution of equivalent plastic strain in the honeycomb core depicted in Figure 9, there are no appreciable differences in the equivalent plastic strain and damage produced by the three materials when they served as the structural components of the honeycomb core during the impact process. Combining the results from Figure 7 and Figure 9, it can be seen that GF-PEEK exhibits greater structural stability as a face sheet material than CF-PEEK.

The face sheet, made of CF-PEEK, exhibits the least stability when subjected to the same impact energy conditions, according to an analysis of the stress and strain on the honeycomb structure from above. Contrary to the initial plan, the carbon fiber-reinforced PEEK material, in this instance, actually makes the honeycomb sandwich construction less stable.

### 3.3. Face Sheet Deformation

**Figure 10:**
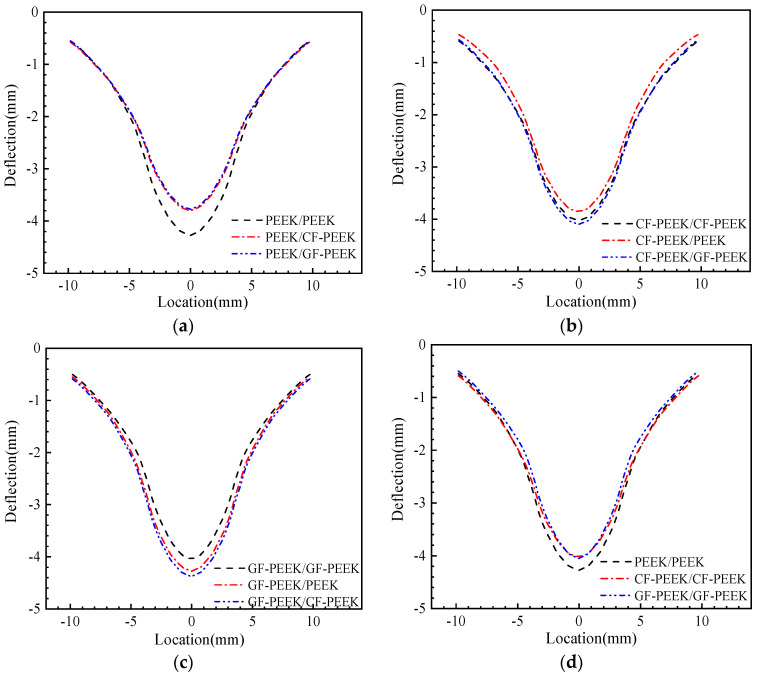
Numerical results for the middle line deflection of the front face sheet (**a**) PEEK panels structure; (**b**) CF-PEEK panels structure; (**c**) GF-PEEK panels structure; and (**d**) comparison of the homogeneous material structure.

The location–deflection curves for various HSS are presented in Figure 10. The front face sheet of the HSS location–deflection curves are symmetrically distributed around the impact axis for different material combinations. The face sheet made of PEEK that covers the honeycomb sandwich construction can be seen in Figure 10a. With a maximum deflection of 4.29 mm, PEEK is the honeycomb core material that exhibits the most deflection in the front face sheet of the honeycomb structure. However, when the honeycomb core material is fiber-reinforced PEEK, the face sheet can deflect no more than 3.80 mm. Figure 10b shows the honeycomb sandwich structure with CF-PEEK as the core material, which results in the highest deflection of the front face sheet. In contrast, the use of PEEK as the core material results in the least amount of deflection, with both the minimum and maximum deflections being 3.88 and 4.05 mm, respectively. The HSS in Figure 10c is made with GF-PEEK, which is used to construct both the front and rear panels. Honeycomb core materials of GF/PEEK exhibit the least face sheet deflection compared to other honeycomb core materials. When CF-PEEK is used as the honeycomb core material, the face sheet deflects the most, with a minimum deflection of 4.06 mm and a maximum deflection of 4.40 mm, respectively. These results suggest that the honeycomb core material significantly impacts the deflection of the front face sheet in HSS. The use of fiber-reinforced PEEK as the core material can effectively reduce the deflection of the front panel. These findings are consistent with previous studies on the mechanical properties of HSS [44,48]. In addition, the comparison of the deflection of the front face sheet of HSS made from the same material is presented in Figure 10d. It can be observed that the use of fiber-reinforced PEEK material results in the minimum front panel deflection, while the use of PEEK material results in a maximum deflection of 4.29 mm.

Based on the preceding analysis, it can be concluded that the glass dimension-reinforced PEEK material as a face sheet of the HSS structure has an opposing adverse effect detrimental to its stability. As shown in Figure 10d, the addition of fiber to PEEK material can significantly improve the deformation resistance of honeycomb structures.

### 3.4. Energy Absorption

**Figure 11:**
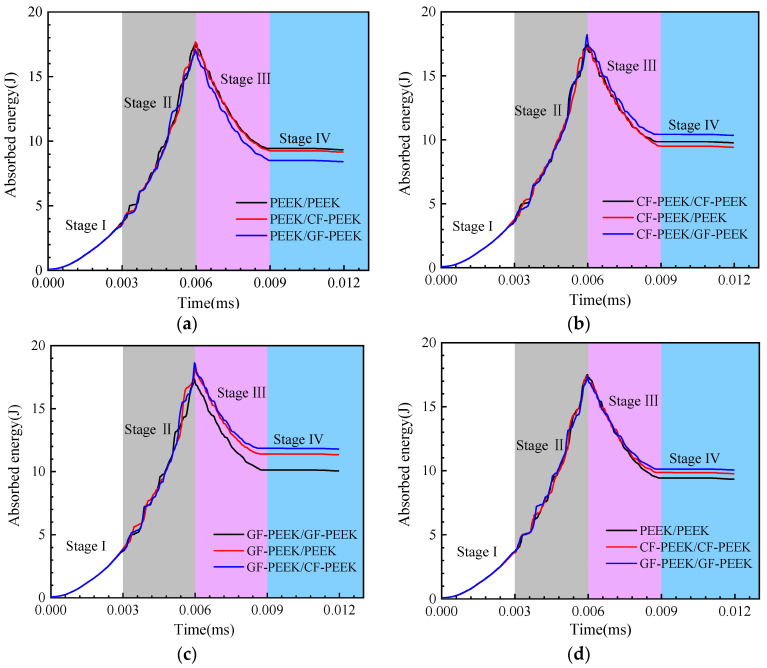
Absorbed energy versus time curves for the composite honeycomb sandwich structure (**a**) PEEK panels structure; (**b**) CF-PEEK panels structure; (**c**) GF-PEEK panels structure; and (**d**) comparison of the homogeneous material structure.

The absorbed energy versus time curves for the HSS were analyzed and divided into four stages. In stage I, the HSS exhibited elastic deformation. In stage II, there was a linear growth phase where the plastic strain was mainly concentrated in the front face sheet and honeycomb core. In stage III, the plastic deformation stage followed the fracturing of the front face sheet. In stage IV, the impactor rebounded, and the contact force rapidly decreased. The graphs presented in Figure 11 illustrate that as the impact time increased, the absorbed energy of all specimens grew to the specified impact energy and subsequently showed a modest decline. The decrease in absorbed energy was due to the transformation of the specimens’ elastic potential energy into the impactor’s kinetic energy, causing the drops to match the impactor’s rebound. By comparing Figure 11a,b and Figure 11a, it is evident that the face sheet material with PEEK as the composite HSS absorbs the least impact energy, while GF-PEEK material absorbs the most impact energy. Additionally, it can be seen that PEEK, which makes up the composite honeycomb sandwich structure, has the lowest absorption energy during the impact process when the three materials in Figure 11d are contrasted as the face sheet and core materials of the structure simultaneously. These findings suggest that the choice of materials for the face sheet and core of the HSS significantly impacts its energy absorption during impact events. The use of GF-PEEK material as the face sheet and core material can effectively enhance the energy absorption of the HSS, which is consistent with previous studies on the mechanical properties of composite materials [49].

## 4. Conclusions

This study conducts a finite element analysis of the low-velocity impact response is conducted for HSS made of various material combinations. The results can be concluded as follows:

(1) The load–displacement curves of several material types as composite HSS are studied, and it is demonstrated that fiber-reinforced PEEK material significantly increases the overall structure’s impact resistance. It is discovered that GF-PEEK is better suited as a face sheet for the composite honeycomb sandwich construction; under the same impact energy, its damage degree is 1.05% and 3.20% lower than those of PEEK and CF-PEEK as panel materials, respectively;
(2) By comparing the plastic deformation of the honeycomb core and the stress and strain distribution of the composite honeycomb sandwich mechanism after low-velocity impact, it is observed that the CF-PEEK face sheet causes more visible damage to the honeycomb core, resulting in more obvious plastic deformation;
(3) The disturbance caused by fiber-reinforced material is compared to the deflection caused by low-velocity dynamic impact, and the use of fiber-reinforced PEEK material results in the minimum front panel deflection, while the use of PEEK material results in a maximum deflection of 4.29 mm. It is more noticeable when the face sheet and honeycomb core of the HSS are made of the same fiber-reinforced PEEK material. The maximum deflection is 4.05 mm and 4.40 mm for GF-PEEK and GF-PEEK, respectively.

These results suggest that the choice of material combination significantly impacts the impact resistance and structural stability of the honeycomb sandwich structure. The findings provide insights into the design and optimization of composite HSS for impact resistance applications.

## Figures and Tables

**Table 1:** Material parameters of the nylon composites [39,40,41].

Material Property	PEEK	CF-PEEK	GF-PEEK
*ρ* (g/cm^3^)	1.36	1.60	1.51
*E* (GPa)	3.85	22	14.2
*v*	0.30	0.33	0.298
Yield strength (MPa)	107	94	87

**Table 2:** A summary of the specimens’ material distribution of the composite honeycomb sandwich structure.

Specimen Number	Face Sheet Material	Honeycomb Core Material
Specimen 1	PEEK	PEEK
Specimen 2		CF-PEEK
Specimen 3		GF-PEEK
Specimen 4	CF-PEEK	CF-PEEK
Specimen 5		PEEK
Specimen 6		GF-PEEK
Specimen 7	GF-PEEK	GF-PEEK
Specimen 8		PEEK
Specimen 9		CF-PEEK

## Data Availability

Not applicable.
